# Comprehensive characterization of ligand‐induced plasticity changes in a dimeric enzyme

**DOI:** 10.1111/febs.13788

**Published:** 2016-07-07

**Authors:** Oliver M. Baettig, Kun Shi, Brahm J. Yachnin, David L. Burk, Albert M. Berghuis

**Affiliations:** ^1^ Department of BiochemistryGroupe de Recherche Axé sur la Structure des ProtéinesMcGill UniversityMontrealQCCanada; ^2^ Department of Chemistry & Chemical BiologyCenter for Integrative ProteomicsRutgers UniversityPiscatawayNJUSA; ^3^ Department of Microbiology and ImmunologyGroupe de Recherche Axé sur la Structure des ProtéinesMcGill UniversityMontrealQCCanada

**Keywords:** allosteric cooperativity, aminoglycoside 6′‐*N*‐acetyltransferase type‐Ii, antibiotic resistance, ligand‐induced plasticity, protein flexibility

## Abstract

An enzyme's inherent structural plasticity is frequently associated with substrate binding, yet detailed structural characterization of flexible proteins remains challenging. This study employs complementary biophysical methods to characterize the partially unfolded structure of substrate‐free AAC(6′)‐Ii, an *N*‐acetyltransferase of the GCN5‐related *N*‐acetyltransferase (GNAT) superfamily implicated in conferring broad‐spectrum aminoglycoside resistance on *Enterococcus faecium*. The X‐ray crystal structure of AAC(6′)‐Ii is analyzed to identify relative motions of the structural elements that constitute the dimeric enzyme. Comparison with the previously elucidated crystal structure of AAC(6′)‐Ii with acetyl coenzyme A (AcCoA) reveals conformational changes that occur upon substrate binding. Our understanding of the enzyme's structural plasticity is further refined with small‐angle X‐ray scattering and circular dichroism analyses, which together reveal how flexible structural elements impact dimerization and substrate binding. These results clarify the extent of unfolding that AAC(6′)‐Ii undergoes in the absence of AcCoA and provide a structural connection to previously observed allosteric cooperativity of this enzyme.

**Database:**

Structural data are available in the PDB database under the accession number 5E96.

**Abbreviations**
AAC(6′)‐Ii: aminoglycoside 6′‐*N*‐acetyltransferase type‐Ii
AcCoA: acetyl coenzyme A
CD: circular dichroism
GNAT: GCN5‐related *N*‐acetyltransferase
NMR: nuclear magnetic resonance
RMSD: root‐mean‐square deviation
SAXS: small‐angle X‐ray scattering

## Introduction

Motion due to protein plasticity frequently links a protein's structure to its function and acts as an important determinant of a protein's intrinsic properties such as stability. Macromolecular motions may span a large time range and distance and thus cannot be accurately represented by any single biophysical technique, each of which informs on dynamic processes of a limited time scale and resolution 1. Although enzymes are often characterized as relatively rigid, partial conformational changes are frequently a condition for catalysis. Local plasticity such as domain reorientations, pivots about hinge loops, and reordering of amino acid side chains to form or break noncovalent contacts often constitute essential motions in enzymes or enzyme‐complexes 2. Such large amplitude collective motions typically occur between a small number of kinetically distinguishable states, and common methods describing such conformational motion include X‐ray crystallography, nuclear magnetic resonance (NMR), small‐angle X‐ray scattering (SAXS), circular dichroism (CD), and fluorescence spectroscopy. In contrast, a locally disordered portion of an enzyme can fluctuate among a large ensemble of structures and traditional biophysical techniques give way to computational methods in this range 3.

In this study, we use a combination of CD, SAXS, and X‐ray crystallography to investigate the structure of the aminoglycoside 6′‐*N*‐acetyltransferase type‐Ii (AAC(6′)‐Ii). This aminoglycoside detoxifying enzyme is a single‐domain, dimeric protein found in *Enterococcus faecium* 4 and purportedly contributes to this bacterium's broad resistance profile. The kinetic mechanism and the CoA as well as acetyl coenzyme A (AcCoA)‐bound crystal structures of AAC(6′)‐Ii have been previously reported, yet the apoprotein (apo) structure remains elusive 5, 6, 7, 8. Structures of AAC(6′)‐Ii in complex with a CoA or AcCoA‐group both show a V‐shaped fold consisting of two sets of antiparallel β‐sheets surrounded by α‐helices, a fold typical of proteins belonging to the GCN5‐related *N*‐acetyltransferase (GNAT) superfamily. Members of the GNAT superfamily all utilize the cofactor AcCoA and share a strongly conserved core structure despite minimal sequence similarities 9, 10. The catalytic cycle of AAC(6′)‐Ii follows an ordered bi‐bi mechanism, where AcCoA binds prior to the aminoglycoside substrate and CoASH departs after the acetylated product 7.

The present work builds on our mechanistic characterization of AAC(6′)‐Ii 11, 12, which was largely based on NMR and ITC studies. These solution studies probed the cooperativity of AcCoA binding between the two protomers of the dimeric enzyme and indicated that the apo form was much more flexible than the substrate‐bound form. This finding is consistent with early protease analyses and heteronuclear single quantum coherence studies 8. Indeed, AcCoA binding potentially induced significant structural changes in the enzyme. We address here two questions that could not be satisfactorily answered previously, namely whether the apo form is partially disordered, and how exactly the dimer interface is affected upon acetyl‐CoA binding. By combining results from different structural biology methods, we propose a nuanced model of the conformational flexibility of AAC(6′)‐Ii upon substrate binding.

## Results

### X‐ray crystallography

**Figure 1:**
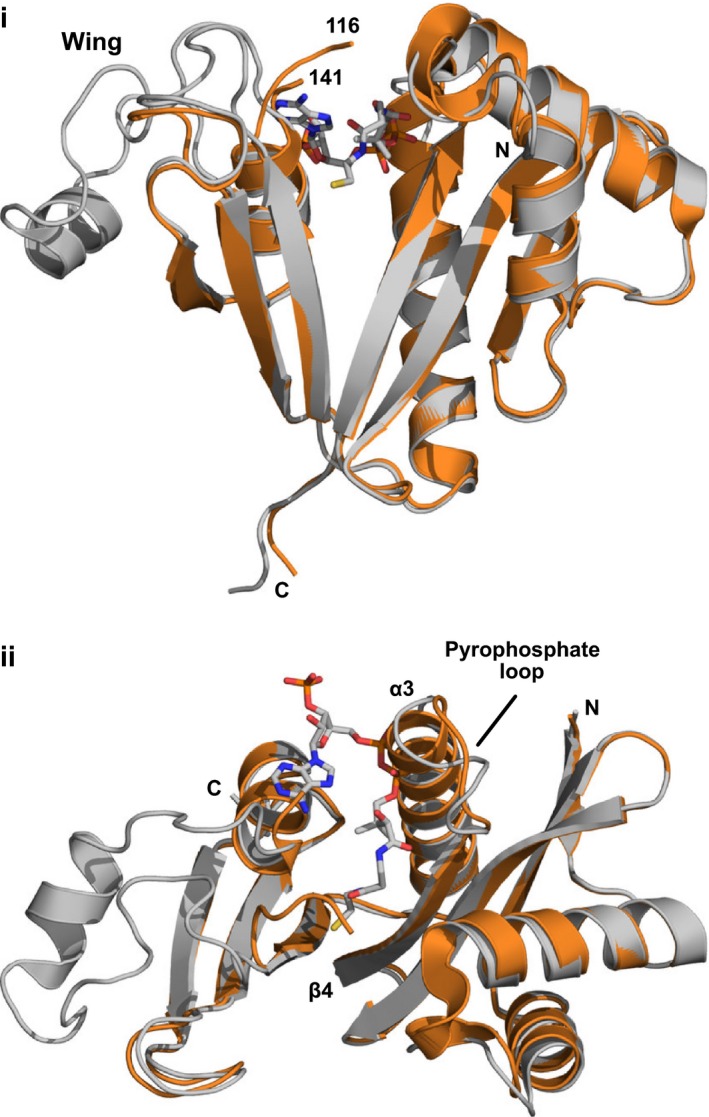
Crystal structure of apo AAC(6′)‐Ii. (i) Cartoon representation of apo AAC(6′)‐Ii (orange) superimposed onto one monomer of the AAC(6′)‐Ii‐AcCoA binary complex (gray with ligand in stick representation, PDB code 1N71). While most structural elements are conserved, a flexible wing‐shaped region (residues 117–140) and the pyrophosphate loop (residues 80–86) show clear differences. (ii) Cartoon representation of apo AAC(6′)‐Ii, as above but rotated 90° about an axes running horizontally through the center of the page. This view illustrates how residues of the pyrophosphate loop of the apo structure adopt a helical conformation and would occupy the same space as the pyrophosphate of the coenzyme.

The apo AAC(6′)‐Ii crystal structure has been determined and refined in space group P6_4_ with one protein molecule per asymmetric unit to 2.1 Å with an *R*_cryst_ of 0.217 and an *R*_free_ of 0.256. Although previously reported NMR data indicate that the apo form of AAC(6′)‐Ii is highly flexible 8, the X‐ray crystallographic analysis shows a mostly well‐structured protein. In comparison to the previously obtained binary complexes, the apo structure shares an identical overall fold and retains 90% of secondary structural features. However, residues 117–140 and residues 80–86 of the apo structure diverge significantly from the substrate‐bound structures. Residues 117–140 could not be accurately modeled due to poor electron density, indicating a highly flexible local structure (Fig. 1). The presence of an adjacent solvent channel permits flexibility in this region, and partially accounts for the high solvent content of 70% observed for this crystal. In contrast, in the substrate‐bound structures, these residues form an ordered wing‐like structure that contains a single helix. The second structural difference lies in what has previously been named the pyrophosphate loop between β4 and α3 (residues 80–86) and is likely a result of the missing substrate. In the AcCoA‐bound structure, this loop is more extended to allow for binding of the pyrophosphate group of acetyl‐CoA. In the absence of substrate, residues 83–86 extend helix α3 and partially occupy the acetyl‐CoA‐binding site (Fig. 1).

Although AAC(6′)‐Ii functions as a physiological dimer, its crystal structure has only one protein molecule per asymmetric unit as the symmetry axis relating the two protomers coincides with a crystallographic symmetry axis in this crystal form. An analogous situation was also previously observed for one of the CoA‐bound crystal structures 5. It should be noted that a portion of the missing wing‐like region (residues 124–133) is implicated in forming the dimer interface in the complexed or binary structures. We propose that the main hydrophobic interactions afforded by these residues stabilize dimerization, but are nonessential as this wing‐like region is unique to AAC(6′)‐Ii among members of the GNAT superfamily, many of which also form physiological dimers 6.

**Figure 2:**
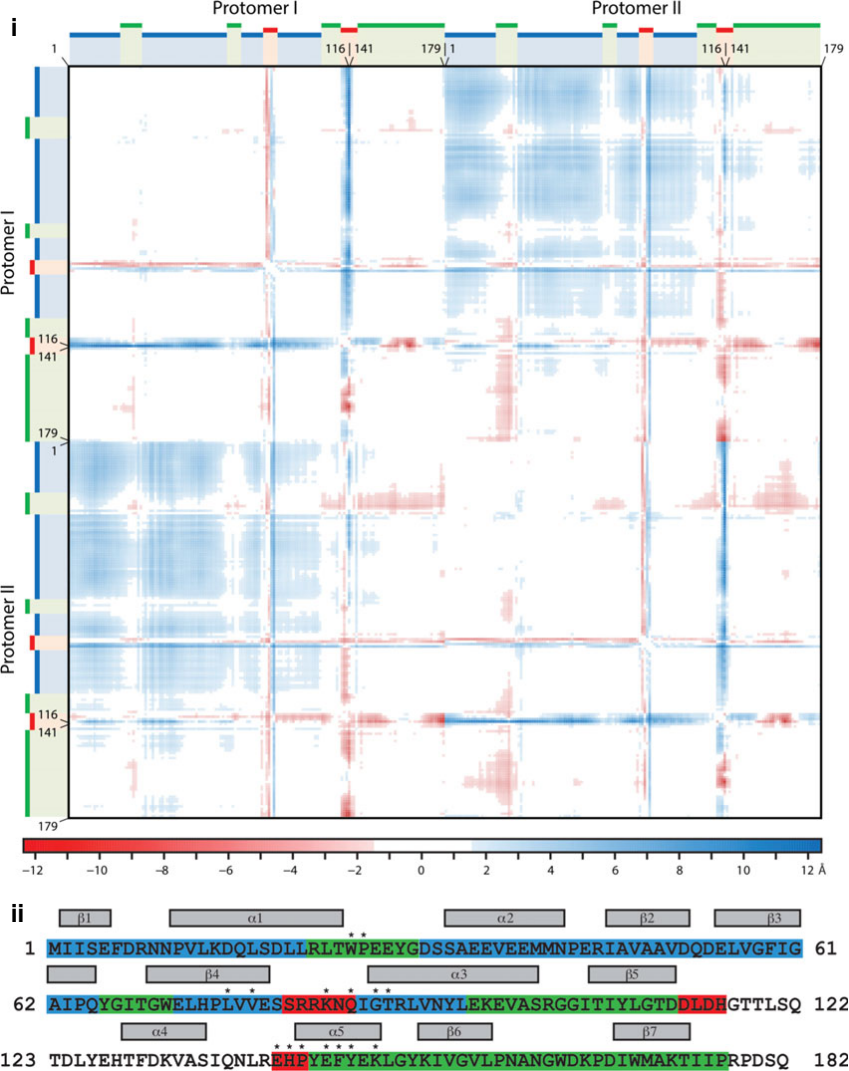
Difference distance matrix contrasting apo with binary state. (i) The difference distance matrix compares the reconstituted dimer of the apo form to the binary complex (SegID A & B of 1N71) with a normalized RMSD of 1.4 Å, with residues 117–140 removed to account for the missing segment of the apo form. Difference values are plotted by color, where light to dark red represents differences of −1.4 to −12 Å, light to dark blue represents 1.4–12 Å, and white represents values between −1.4 and 1.4 Å. Green bars highlight positionally conserved residues (22–30, 66–71, 105–112, 144–179), red bars highlight positionally distinct residues (81–86, 113–116, 141–143), and blue bars highlight residues that are different when comparing distances across the dimer, i.e., distance of protomer II in relation to protomer I, but not within the same protomer (1–21, 31–65, 72–80, 87–104). (ii) Amino acid sequence of AAC(6′)‐Ii, color‐coded according to positional conservation as determined by the difference distance matrix.

**Figure 3:**
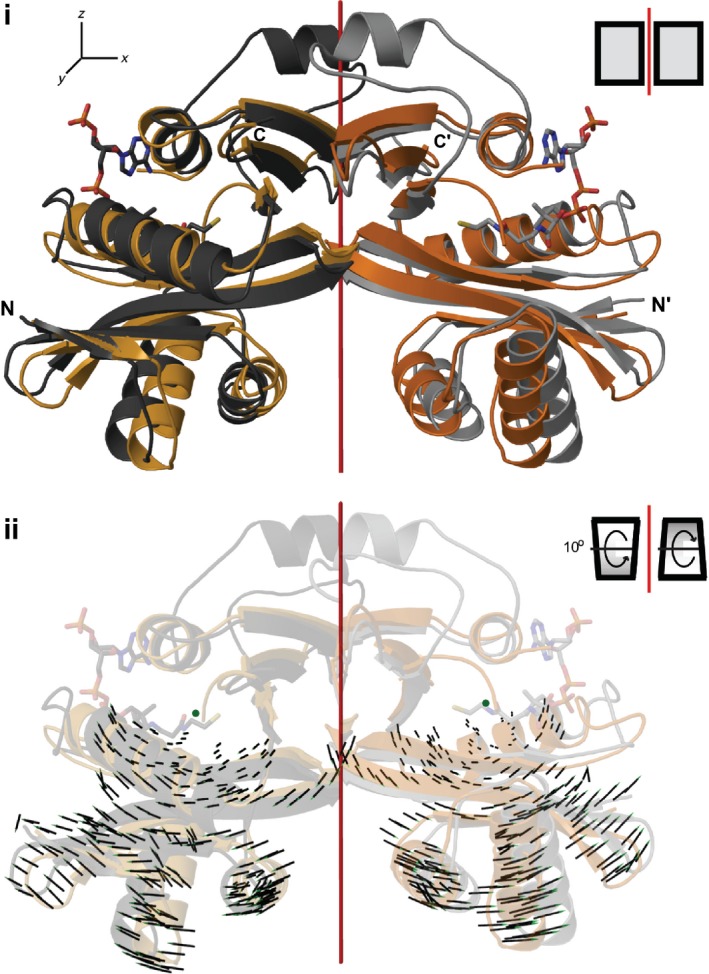
Superposition of the reconstituted apo dimer with the dimer of the AAC(6′)‐Ii‐AcCoA binary complex. (i) The dimer of the apo form (orange) overlaid with the dimer of the binary complex (gray) illustrates the conformation shifts in the N‐terminal portion of AAC(6′)‐Ii (dimer axis shown in red). (ii) The rotational movement of each N‐terminal lobe is highlighted by vectors between corresponding α‐carbons of the apo and binary AAC(6′)‐Ii structures. In order to align the rotational axes (green dot) of both protomers perpendicularly with the page, the planes of each protomer were rotated by 10° in opposing directions along the *x*‐axis.

Using an apo AAC(6′)‐Ii dimer that spans two asymmetric units, a difference distance matrix was generated to analyze any structural differences with the AcCoA‐bound form of AAC(6′)‐Ii (Fig. 2i). The overall root‐mean‐square deviation (RMSD) for 179 corresponding backbone atoms was 2.2 Å, with a normalized RMSD for 100 amino acids of 1.4 Å. Not surprisingly, the residues showing the greatest positional changes correspond to those flanking the missing C‐terminal region and those forming the pyrophosphate loop, with displacements up to 12 Å. The difference distance matrix also reveals a series of residues in the N‐terminal portion of the protein (highlighted in blue in Fig. 2ii) that shift in concert by 3–6 Å between the apo and the binary structure. This shift is only observed when comparing the two protomers in the dimeric enzyme and not observed within either protomer, thus implying a conformational difference in the dimer structure when comparing the apo state with the complexed state. This structural shift is illustrated by the superposition of the complexed dimer structure on the apo dimer structure (Fig. 3i). When vectors between corresponding backbone atoms are mapped, a rotation in the N‐terminal lobe of each protomer can be detected (Fig. 3ii). The relative rotation of the protomers can be deconstructed into two components: although the lateral rotation of each N‐terminal lobe away from the dimer axis is only about 3–4°, each protomer also rotates by 10° perpendicular to the dimer axis. Together, these rotations lead to local displacements of up to 6 Å for individual residues in the N‐terminal region.

To further characterize the apparent flexibility of the apo AAC(6′)‐Ii structure suggested by NMR and X‐ray crystallography 11, we utilized additional solution‐based methods to obtain a comprehensive model of the enzyme's flexibility in this apo state.

### Circular dichroism

Recognizing that the pH at which apo AAC(6′)‐Ii crystallizes in this study is at least two units lower than previously determined crystallization conditions, CD spectroscopy was used to probe changes in secondary structure between apo and complexed states of AAC(6′)‐Ii and to explore how different pH conditions might affect those structural elements.

**Figure 4:**
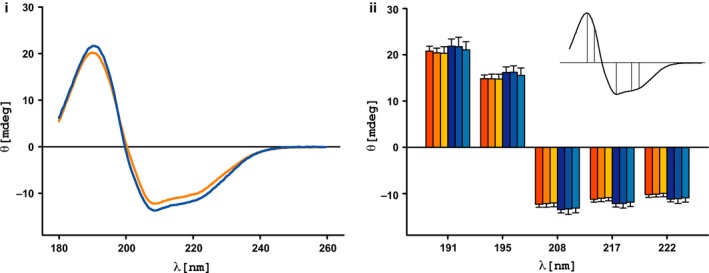
Circular dichroism spectra of apo and complexed AAC(6′)‐Ii. (i) The CD spectrum of apo AAC(6′)‐Ii (blue curve) is superimposed onto that of the protein in complex with AcCoA (orange curve). (ii) Four replicates at three pH levels (pH 5, 6, and 7) were recorded for the enzyme in both apo (three shades of orange) and complexed states (three shades of blue). The molar ellipticity (θ) at each pH level is plotted at five distinct wavelengths of the CD spectrum (191, 195, 208, 217, 222 nm). Error bars denote two standard deviations from the mean.

CD spectra of the apo state at pH 5, 6, and 7 superposed perfectly, indicating no changes in the enzyme's secondary structure composition in this pH range (Fig. 4i). Results of AcCoA‐bound protein samples duplicated this trend, thus we conclude that the secondary structure composition of AAC(6′)‐Ii is pH‐independent in the range at which it crystallizes. In addition, comparison between CD spectra of the apo and complexed forms show no statistically significant differences (Fig. 4ii), indicating that secondary structures present in the apo form remain intact in the complexed form.

We determined the melting temperatures of AAC(6′)‐Ii by measuring the disappearance of alpha helices and beta sheets and the accumulation of random coils over time. Unfolding was reversible, with an average melting temperature for the apo form of 50 °C, while that of the AcCoA‐bound form was 52 °C. The comparable melting temperatures suggest that AcCoA is not necessary to maintain the structural stability of the protein. However, as the experiments were performed with a single rate of temperature increment, the potential contribution of the kinetics of unfolding cannot be excluded. These findings are consistent with our X‐ray crystallographic results, and we conclude that while substrate binding does not improve protein stability, the flexible wing‐like region also does not significantly impact the stability of the apo state.

### SAXS analysis

**Figure 5:**
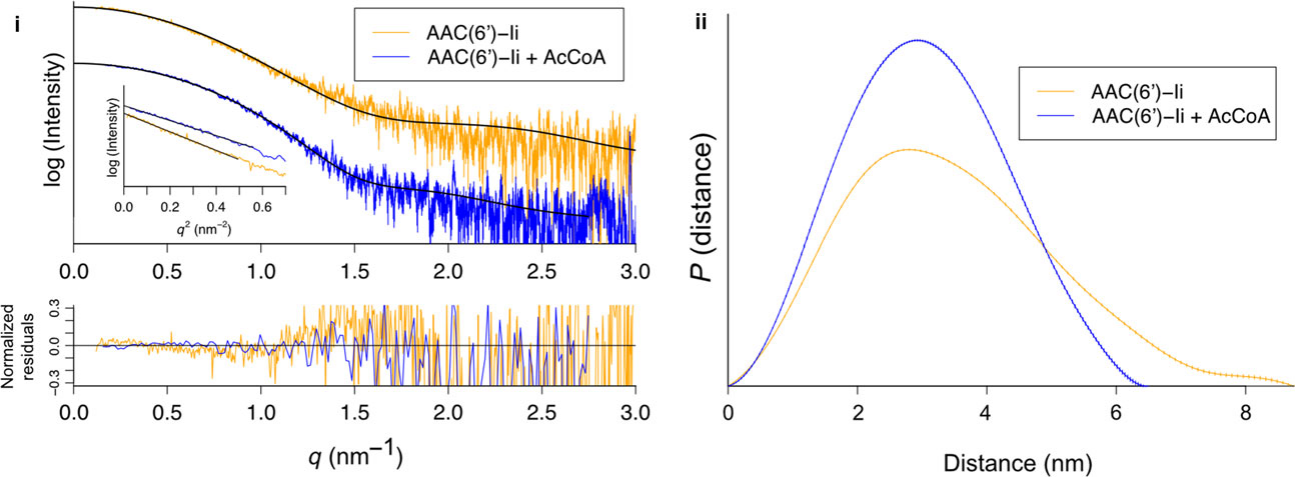
Small‐angle X‐ray scattering curves and corresponding pair‐distribution functions of apo and complexed AAC(6′)‐Ii. Processed scattering curves (i) and corresponding P(r) plots (ii) of the AAC(6′)‐Ii in apo (orange) and complexed states (blue). Error bars are shown as vertical lines. In panel (i), the Guinier plots are shown inset, and the best model (BUNCH rigid body model for the apo state, and crystal structure PDB 1N71 for the complexed state) is shown as a black line. The normalized residuals for those models are shown below the main graph. See also Table 1.

**Figure 6:**
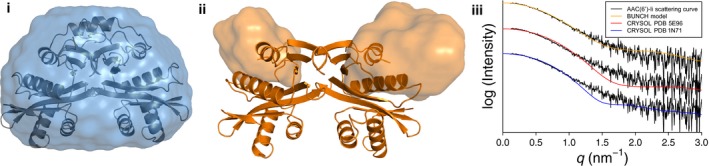
Comparison of the SAXS models for apo and complexed AAC(6′)‐Ii. (i) The *ab initio* model of AAC(6′)‐Ii is shown as an envelope, with the coordinates of the complexed structure (PDB ID 1N71) superimposed onto the model. (ii) The BUNCH model derived from the apo structure (PDB ID 5E96) is shown, with the flexible region shown as envelopes. (iii) A comparison of the goodness‐of‐fit of the BUNCH model (orange), the apo crystal structure (PDB ID 5E96, red), and the complexed crystal structure (PDB ID 1N71, blue) to the apo SAXS scattering curve (black).

We further characterized the shape and size of the apo and complexed states of AAC(6′)‐Ii in solution using small‐angle X‐ray scattering (SAXS; Fig. 5i). We noted a marked difference between the scattering data of the apo form and the AcCoA‐bound form. The enzyme–substrate complex, which showed good agreement (Table 1, Fig. 6i) with the crystal structure (PDB ID 1N71), exhibited a lower radius of gyration and a maximum diameter that measured 20 Å shorter than the apo protein (Table 1, Fig. 5ii). The bell‐shaped pair‐distribution function of the substrate‐bound form is indicative of a roughly spherical overall shape, with a maximum diameter of 65 Å, which agrees well with the measured value of 67 Å from its crystal structure. On the other hand, the asymmetric curve for the apo form suggests that in this state, AAC(6′)‐Ii departs from the spherical shape, with at least one dimension at 85 Å, or 30% wider than the maximum diameter of the complexed structure. These results indicate that AAC(6′)‐Ii exhibits a more compact shape when bound to AcCoA than in its absence. We propose that this difference can be attributed to the flexible wing‐like region (residues 117–140) that is missing from the apo crystal structure. Using the atomic coordinates of the apo structure in conjunction with the entire protein sequence, we reconstructed a complete apo model containing the missing residues in the C‐terminal region using BUNCH (Fig. 6ii) 13. The resulting model has a spherical core with wing‐like protrusions corresponding to the missing residues, and presents a markedly improved fit to the original scattering curve compared to either the substrate‐complexed crystal structure (PDB ID 1N71) or the apo crystal structure presented here (Fig. 6iii). Though by no means an accurate atomistic model of apo AAC(6′)‐Ii, we believe that this rigid body reconstruction model confirms the presence of a flexible region in the C‐terminal lobe of the enzyme and provides a rationale for the difference in radius of gyration between the apo and complexed states.

**Table 1:** SAXS statistics

	AAC(6′)‐Ii	AAC(6′)‐Ii + AcCoA
Guinier R_g_ (Å)	26.5 ± 0.1	23.0 ± 0.1
Real space R_g_ (Å)^a^	26.8 ± 0.08	23.3 ± 0.03
Guinier *I*(0)	0.11000 ± 0.00021	0.13000 ± 0.00022
Real space *I*(0)^a^	0.1133 ± 0.0002	0.1354 ± 0.0001
*D*_max_ (Å)^a^	87.5	65
Total^a^	0.691	0.688
crysol χ (PDB ID 1N71)	2.802	1.606
crysol χ (PDB ID 5E96)	3.303	1.711
BUNCH χ	2.267	N/A

^a^ Calculated by gnom 19.

## Discussion

**Figure 7:**
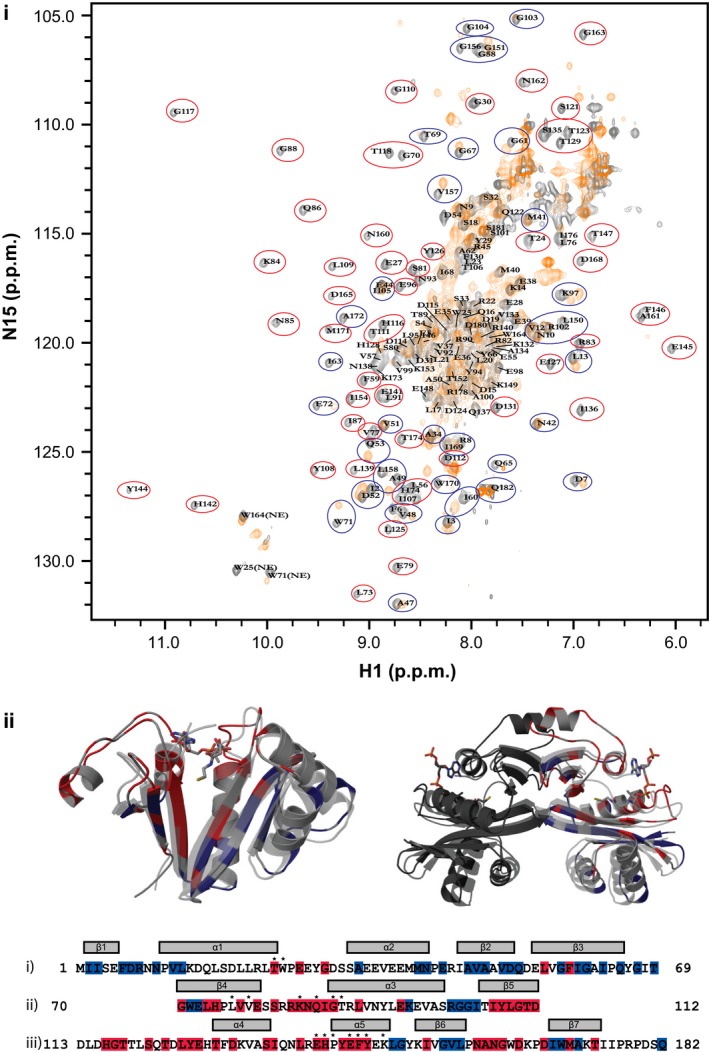
Comparison of HSQC spectra of apo state and binary complex. (i) ^15^N/^1^H HSQC spectrum of the apo state (orange) is superimposed on AcCoA binary complex (black). Blue circles indicate residues with resolved peaks in both spectra. Red circles indicate single peaks in the binary complex spectrum. (ii) Comparison of the HSQC data with crystal structure (monomer left; dimer right; apo state transparent; and binary complex solid) and AAC(6′)‐Ii sequence divided into (i) N‐terminal region; (ii) central region; and (iii) C‐terminal region. The coloring of the residues corresponds to the HSQC spectra. The * indicates residues interacting with AcCoA. Note that these data have been previously reported by Freiburger *et al*. 11.

While previous NMR HSQC studies of AAC(6′)‐Ii suggested a mostly flexible apo form 11, our crystal structure of this form indicates that it largely retains its well‐ordered structure. Combining the results of different biophysical techniques allowed us to provide a more rigorous description of the apo structure and qualify the extent of the plasticity that AAC(6′)‐Ii exhibits in the apo form. The enzyme retains most of its secondary structural elements regardless of AcCoA binding. In particular, the mixed β‐sheet that forms the core of the enzyme remains intact according to our CD analyses. In addition, results from SAXS studies indicate that upon AcCoA binding, a large, flexible region becomes more structured, which we attribute to the 24‐residue long, wing‐like region in the C‐terminal lobe (residues 117–140) that is unstructured in the apo crystal structure. To complement previous NMR findings, EXSY experiments have been used to link 38 apo peak shifts with known peaks of the complexed form's spectrum (Fig. 7) 11. Given that the wing‐like region is disordered in the apo form, we can expect that amide peaks from these residues would locate in the central area of the apo HSQC spectrum, which has been confirmed for three residues (Ser121, Asp131, and Ile136). We are now in a position to refine the hypothesis that the apo form of AAC(6′)‐Ii is highly flexible. We suggest that the quaternary structural changes induced by AcCoA binding include a bulk movement of the N‐terminal regions away from each other as well as an ordering of residues 117–140 leading to the formation of a more extensive dimer interface. Despite these differences, the overall structure of apo AAC(6′)‐Ii mirrors the binary form relatively well and does not undergo large‐scale conformational changes or significant unfolding as suggested by previous NMR studies 11.

It remains unclear what function, if any, the flexible region in the C‐terminal lobe fulfills. As it is not directly involved in AcCoA or aminoglycoside binding, it is tempting to speculate that it may have an allosteric sensing function that regulates enzyme activity. In this respect, it is worth noting that the antibiotic resistance conferring function may not be the primary task performed by AAC(6′)‐Ii, though its native role has remained elusive 14. Given that the wing‐like region is unique to AAC(6′)‐Ii among GNAT family members, its specific order–disorder transition upon the presence/absence of AcCoA suggests that it could potentially act as a recognition site for cell signaling.

An allosteric binding model consisting of a hybrid of the Koshland–Nemethy–Filmer and the Hilser–Thompson (HT) models has been proposed for AAC(6′)‐Ii 11. In the Koshland–Nemethy–Filmer model, free and bound subunits assume distinct conformations, conformational changes only occur upon substrate binding, and cooperativity results from inter‐subunit interactions 15. In the HT model, allostery is driven by coupled folding and binding of adjacent protomers, which explicitly includes conformational equilibria in unbound subunits 16. Thermodynamic studies using ITC, CD, and NMR have indicated that each protomer of the enzyme can exist in at least three conformational states, namely a partially unfolded, a free‐folded, and a substrate‐bound or completely folded state, though no structural descriptions of the substrate‐free states could be furnished. The addition of the apo crystal structure complements our understanding of the enzyme's allosteric binding model. We hypothesize that the partially unfolded state corresponds to the apo crystal structure described here, with the flexible region of the C‐terminal lobe unstructured as suggested by the SAXS data. The free‐folded state would thus resemble an enzyme conformation where the wing‐like region is folded, while the pyrophosphate loop remains in its alternative conformation as no AcCoA binding has occurred yet. Thus, there exist two distinct apo structures. This would imply that firstly, in a substrate‐binding event, the flexible region (residues 117–140) folds before the pyrophosphate loop, and secondly, the free‐folded form is highly transient and less stable than both the partially unfolded and the substrate‐bound state. Alternatively, the apo structure determined here represents the free‐folded structure and that there still exists another, less structured, conformation of the apo state that is perhaps too disordered for crystallization. We consider this possibility unlikely as results from the CD studies give no evidence of another conformation with less secondary structure and more random coil. Positive cooperativity may be a result of the dimerization interaction of the flexible region in the C‐terminal lobe. With one AcCoA molecule bound and thus one protomer fully folded, as the second protomer shifts toward the free‐folded state, the formation of the structured C‐terminal wing‐like loop causes N‐terminal portions of the two protomers to undergo a rotational inward shift which tightens the V‐shaped cleft (Fig. 3i). Considering that binding interactions between AcCoA and AAC(6′)‐Ii occur predominantly via backbone hydrogen bonds, this slight rotation may significantly facilitate the binding of the second AcCoA substrate, thus leading to cooperativity.

In summary, through a combination of X‐ray crystallography, CD, SAXS, and NMR, we determined the structure of the apo conformation of AAC(6′)‐Ii and characterized its plasticity. We pinpointed the flexible regions of the apo state to the pyrophosphate loop and a section of the C‐terminal lobe, and showed that substrate binding involves the ordering of these two loops as well as a rotational shift of the N‐terminal portion of each protomer of the catalytically active dimer. These results not only clarify the nature and extent of the apo state's flexibility but also allow us to propose a structural mechanism for an intriguing allosteric binding model for this enzyme which incorporates simultaneous positive and negative cooperative mechanisms.

## Experimental procedures

### Crystallization, data collection, and refinement

AAC(6′)‐Ii was expressed and purified as previously described 14. The apo crystal of AAC(6′)‐Ii was obtained by equilibrating a 4‐μL drop consisting of 2 μL of protein at 7 mg·mL^−1^ solubilized in 25 mm HEPES pH 7.5 and 2 mm EDTA and 2 μL of reservoir solution (40% ethylene glycol, 0.1 m phosphate–citrate pH 4.2, 0.1 m ammonium sulfate) against 1000 μL of the reservoir solution at 4 °C via the hanging drop vapor diffusion method. Diffraction data were collected on beamline X6A at the National Synchrotron Light Source for 164 frames with a 1° oscillation angle. The crystal structure of apo AAC(6′)‐Ii was solved though Phaser using molecular replacement with the binary AAC(6′)‐Ii (PDB code: 1N71) as the search model. For the molecular replacement, AAC(6′)‐Ii was truncated to the core secondary structures common to all GNAT family members to calculate a preliminary electron density map. Subsequent refinement cycles alternated between manual model building and reciprocal space refinement. See Table 2 for a summary of key data collection and refinement parameters.

**Table 2:** Data collection and refinement statistics for apo AAC(6′)‐Ii

	APH(2″)‐IVa
Resolution range (Å)	50.0–2.1 (2.18–2.10)^a^
Space group	P6_4_
a, b, c (Å)	66.82, 66.82, 104.13
α, β, γ (°)	90, 90, 120
Number of unique reflections	14 868
Completeness (%)	99.9 (100)
Redundancy	9.6 (9.4)
Mean *I*/σ(*I*)	38.7 (11.2)
*R*_sym_	0.033 (0.206)
*R*_cryst_ b/*R*_free_ c	0.217/0.256
Number of nonhydrogen atoms	
Protein	1246
Ligand	9
Solvent	53
RMSD	
Bond lengths (Å)	0.006
Bond angles (°)	1.343
Average thermal factor (Å^2^)	
Protein	41.9
Substrate	56.9
Solvent	45.9

^a^ Values in parentheses refer to reflections in the highest resolution shell. ^b^ *R*_cryst_ = Σ(|*F*_o_| – |*F*_c_|)/Σ|*F*_o_|, where |*F*_o_| is the observed and |*F*_c_| is the calculated structure factor amplitude of a reflection. ^c^ *R*_free_ was calculated by randomly omitting 5% of the observed reflections from the refinement.

### Circular dichroism

CD measurements were obtained using a ChiraScan CD instrument (Applied Photophysics, Leatherhead, Surrey, UK). Each sample contained 500 μL of protein solution consisting of AAC(6′)‐Ii at 0.5 mg·mL^−1^ and 100 μL of deionized water or 100 μL of 50 μm AcCoA. For measurements at different pH values, the protein solution was prepared in a 5 mm sodium‐phosphate buffer at pH 6 and subsequently mixed at a 1 : 1 ratio with a 50 mm sodium‐phosphate buffer at the desired final pH. The wavelength spectrum used extended from 180 to 260 nm in 0.5 nm steps. For temperature‐dependence studies, AAC(6′)‐Ii at 100 μg·mL^−1^ in sodium‐phosphate buffer at pH 6 in the absence or presence of 20 μm AcCoA was used. Changes in molar ellipticity (θ) were measured at wavelengths 191, 195, 208, 217, and 222 nm, while temperature was increased from 6 °C to 94 °C and then reduced to 6 °C in 2 °C per 2 min intervals.

### Small‐angle X‐ray scattering

Small‐angle X‐ray scattering measurements were performed on a SAXSess small‐angle X‐ray scattering instrument (Anton Paar, Graz, Austria) equipped with a CCD detector (Princeton Instruments, Trenton, NJ, USA). The beam length was set to 18 mm. AAC(6′)‐Ii was concentrated to 10 mg·mL^−1^ and dialyzed against 50 mm sodium‐phosphate buffer pH 6 containing 2 mm EDTA. To obtain the AcCoA‐complexed sample, 5 mm AcCoA was also included in the dialysis buffer. Protein samples were diluted using the dialysis buffer to the desired concentration for data collection. All data collection was performed at 22 °C. About 40 μL protein samples (4–10 mg·mL^−1^) was irradiated over a series of 1440 ten‐second exposures (4‐h exposure time), while dialyzed buffer samples were irradiated over a series of 2880 ten‐second exposures (8 h). Scattering intensities (*I*) were recorded along a two‐dimensional slit and averaged to obtain a one‐dimensional scattering curve with a q‐range of 0.012–0.3 Å^−1^. Dark current correction, normalizing, buffer subtraction, and desmearing were performed in SASQuant 3.0. Independent SAXS measurements were performed at three different protein concentrations, at minimum, and were compared to rule out any concentration‐dependent effects. Subsequent data analysis was performed using the atsas software suite 17. The Guinier analysis was performed in primus 18. Probability distance distribution plots were generated using gnom, and fits to the crystal structures of AAC(6′)‐Ii were calculated using crysol 19, 20. For the complexed dataset, an *ab initio* model of AAC(6′)‐Ii was calculated using gasbor 50 times, and then averaged using damaver, while enforcing P2 symmetry 21, 22. The crystal structure (PDB ID 1N72) was superimposed onto this SAXS envelope. For the apo dataset, in order to model the wing‐like residues in the C‐terminal lobe that are missing from the apo crystal structure (PDB ID 5E96), BUNCH was used 13. In brief, two fixed ‘domains’ were defined corresponding to the residues observed in the crystal structure, connected by a mobile region corresponding to the missing residues. This model was refined against the apo scattering curve in BUNCH, while enforcing P2 symmetry. The SAXS data curves and statistics are presented in Figs 5 and 6, and Table 1.

## Author contributions

OMB participated in the design of the experiments, collected and analyzed the data presented and cowrote the manuscript. KS cowrote the manuscript and assisted with data interpretation. BJY assisted with the analysis and interpretation of the SAXS data and cowrote the manuscript. DLB assisted with data interpretation and cowrote the manuscript. AMB participated in the design of the experiments and cowrote the manuscript.
